# The decline of malaria in Vietnam, 1991–2014

**DOI:** 10.1186/s12936-018-2372-8

**Published:** 2018-06-07

**Authors:** Sandra M. Goldlust, Phung Duc Thuan, Dang Duy Hoang Giang, Ngo Duc Thang, Guy E. Thwaites, Jeremy Farrar, Ngo Viet Thanh, Tran Dang Nguyen, Bryan T. Grenfell, Maciej F. Boni, Tran Tinh Hien

**Affiliations:** 1 Oxford University Clinical Research Unit, Wellcome Trust Major Overseas Programme, Ho Chi Minh City, Vietnam; 2 Department of Biology, Georgetown University, Washington, DC USA; 3 Department of Ecology and Evolutionary Biology, Princeton University, Princeton, NJ USA; 4 National Institutes for Malariology, Parasitology, and Entomology, Hanoi, Vietnam; 5 Centre for Tropical Medicine and Global Health, Nuffield Department of Medicine, University of Oxford, Oxford, UK; 6 The Wellcome Trust, London, UK; 7 Center for Infectious Disease Dynamics, Department of Biology, Pennsylvania State University, University Park, PA USA

**Keywords:** Malaria, Artemisinin, Vietnam, Vector control, Urbanization

## Abstract

**Background:**

Despite the well-documented clinical efficacy of artemisinin-based combination therapy (ACT) against malaria, the population-level effects of ACT have not been studied thoroughly until recently. An ideal case study for these population-level effects can be found in Vietnam’s gradual adoption of artemisinin in the 1990s.

**Methods and results:**

Analysis of Vietnam’s national annual malaria reports (1991–2014) revealed that a 10% increase in artemisinin procurement corresponded to a 32.8% (95% CI 27.7–37.5%) decline in estimated malaria cases. There was no consistent national or regional effect of vector control on malaria. The association between urbanization and malaria was generally negative and sometimes statistically significant.

**Conclusions:**

The decline of malaria in Vietnam can largely be attributed to the adoption of artemisinin-based case management. Recent analyses from Africa showed that insecticide-treated nets had the greatest effect on lowering malaria prevalence, suggesting that the success of interventions is region-specific. Continuing malaria elimination efforts should focus on both vector control and increased access to ACT.

**Electronic supplementary material:**

The online version of this article (10.1186/s12936-018-2372-8) contains supplementary material, which is available to authorized users.

## Background

Over the past 15 years, scale-up in key tools to prevent and treat malaria has contributed to a dramatic reduction in transmission worldwide [1]. Principal among these tools have been insecticide-treated nets, indoor residual insecticide spraying, and artemisinin-based combination therapy (ACT)—the most effective anti-malarial therapy currently available for treatment of uncomplicated *Plasmodium falciparum*. In 2005, the World Health Organization began recommending ACT as first-line therapy for uncomplicated *P. falciparum* [2], although treatment with artemisinin-containing anti-malarials had already begun in areas confronted with resistance to chloroquine, sulfadoxine–pyrimethamine, and mefloquine [3]. Early clinical studies on artemisinin derivatives in the 1990s [4–8] and larger trials of ACT in the 2000s [9–13] demonstrated high clinical efficacy and a rapid killing rate, which would later make ACT the recommended first-line therapy for national malaria control programmes worldwide.

Beyond its well-established clinical efficacy, artemisinin may have population-level benefits in malaria control due to its moderate effect of reducing post-treatment carriage of gametocytes [14–17]—the sexual stage of malaria transmitted from human peripheral blood to *Anopheles* mosquitoes. Treatment of *P. falciparum* with an artemisinin-containing anti-malarial results in rapid killing of parasite asexual stages (99% daily kill rate [18, 19]) and, when combined with a partner drug, results in undetectable parasitaemia by microscopy after 3 days of treatment [8, 20]. Low parasite densities in the blood generally indicate that patients are less likely to transmit gametocytes to mosquitoes [21, 22]. However, the long-term population-level effects of ACT case management on parasite transmission are only now beginning to be documented through retrospective analyses [1, 23, 24], prospective studies [25–28], meta-analyses [29], and mathematical modelling [30–32]. The fact that ACT is typically introduced as a component of comprehensive malaria control efforts makes it challenging to isolate the effectiveness of ACT from that of other concurrently introduced control strategies, such as indoor residual spraying (IRS) and insecticide-treated bed nets (ITNs).

An excellent case study for the long-term effects of artemisinin use in malaria case management is found in Vietnam. Following an epidemic of chloroquine-resistant *P. falciparum* in the late 1980s, Vietnam implemented a new national malaria control programme into which artemisinin-based case management was introduced; vector control practices were expanded and health capacity was strengthened. The incidence of malaria in Vietnam subsequently declined [33, 34]. Data on incidence, anti-malarial use, vector control effort, and various health-systems metrics were recorded in annual reports from Vietnam’s National Institutes for Malariology, Parasitology, and Entomology (NIMPE). An analysis of these data for the provinces in the southern part of the country from 1991 to 2010 showed that the strongest association with reduced malaria incidence was the proportion of stocked or ordered anti-malarial drugs that were artemisinin derivatives [24]. The present study extends the work by Peak et al. [24] by adding data from national-level reports collected including Vietnam’s central and northern provinces, and adding newer annual data from 2011 to 2014. In addition, this study introduces a measure of estimated malaria cases for Vietnam, using the detection and diagnostic capabilities in Vietnam during this time period. The findings of this study support the robustness of the association between adoption of artemisinin-containing anti-malarials for case management of *P. falciparum* and declining malaria incidence.

## Methods

### Data collection

The data used in this analysis were obtained from the Institutes for Malariology, Parasitology, and Entomology (IMPE) located in Ho Chi Minh City and the National Institutes for Malariology, Parasitology, and Entomology (NIMPE) in Hanoi. Data structure and cleaning have been described previously [24]. Briefly, NIMPE is responsible for defining guidelines, training staff, supporting local and provincial malaria posts and clinics, purchasing and distributing anti-malarial drugs, distributing insecticide-treated nets, identifying malaria transmission hot-spots, spraying insecticide in homes, and other control and response efforts [35]. Data from NIMPE/IMPE annual reports from 1991 to 2014 were collected in hard copy for all 58 provinces and 5 municipalities in Vietnam. In order to account for changes in provincial borders over the study time period, some provinces/municipalities were combined for analysis (see Additional file 1).

### Malaria case data and case estimation

Data collected on malaria cases include provincial-level counts of cases, severe cases, and deaths. Malaria case numbers were available as either the number of clinically suspected malaria cases, $y$, or the number of suspected cases confirmed by microscopy, $x$, where $x$ is a subset of $y$ and both may include cases of *P. falciparum* and *Plasmodium vivax* malaria. However, for the $y-x$ cases that remained unconfirmed, the reports do not indicate whether these cases tested negative by microscopy or if they were simply untested. The distinction between cases that tested negative and untested cases could be made if either the positive predictive value, $q$, of clinical diagnosis or the fraction of clinically suspected cases that undergo blood-slide diagnosis, $f_{BSD}$, were known. If $f_{BSD}$ is known, then the total number of suspected cases that undergo microscopy, $y \cdot f_{BSD}$, can be used to calculate the positive predictive value, $q$, where $q$ has a maximum value of one:

$$\begin{aligned} q=\min \left( 1,\frac{x}{y \cdot f_{BSD}} \right) \end{aligned}$$

Likewise, if the positive predictive value is known, then the fraction of suspected cases that underwent blood-slide confirmation can be calculated, unless $q = 1$, in which case all clinically suspected malaria cases are truly malaria. If the rate of blood-slide confirmation, $f_{BSD}$, is known, then the true number of malaria cases (falciparum and vivax combined) can be estimated as:

$$\begin{aligned} x+y \cdot q \cdot (1-f_{BSD}) \end{aligned}$$

which is equal to $\frac{x}{f_{BSD}}$ when $q \le 1$. Since independent estimates of $q$ or $f_{BSD}$ cannot be obtained, $f_{BSD}$ was assumed to increase linearly in each province, with an independent slope for each province, during the years 1991–2014. This assumption is consistent with NIMPE annual reports, which show an increase in health system capacity and microscopy during this period. The linear increase in $f_{BSD}$ was chosen to minimize the variance in the year-to-year positive predictive value, as it is likely that clinicians’ ability to correctly diagnose a malaria case did not change significantly during this time. This assumption is supported by the opinions of IMPE/NIMPE staff. However, the relative prevalence of other febrile diseases is likely to affect the positive predictive value of malaria clinical diagnosis, depending on the level of similarity between the symptoms of these diseases and malaria symptoms.

### Predictor data

Provincial-level annual data on the following potential predictors of provincial malaria case counts in Vietnam from 1991 to 2014 were identified and collected: (1) the proportion of treatment courses for *P. falciparum* containing artemisinin (see Additional file 2), (2) the proportion of the population protected by vector control measures (IRS or ITNs), (3) the proportion of the population living in urban areas (Government Statistics Office, Vietnam), (4) the discretionary budget per capita for the malaria control programme, and (5) staff trained per 100-persons.

Data on vector control measures contained in the annual reports were used to calculate the proportion of the population protected by vector control measures, as has been done in previous work [24]. These data include the total number of people protected by insecticide-treated bed nets and indoor residual spraying, reported as the sum of these two measures. ITN coverage is defined by NIMPE as the proportion of individuals who share an ITN with a maximum of three other household members [36]. Missing data on vector control measures were imputed by linear interpolation.

The reports also contained data on two measures of health system capacity: the discretionary budget and the training of health care workers. Budgetary data, broken down into sub-budget allocations for the local malaria control programme, and data on staff trained to support the malaria control programme, were both available in the reports by province and year. The discretionary budget per capita, which excludes the allocated budget for purchasing anti-malarial drugs, insecticides, and subsidies for ITNs and IRS, were used as measures of health system capacity after adjusting for historic inflation in the VND. The number of staff trained per capita was also used as a measure of health system capacity. Data for these two measures of health system capacity were only available for the years 1997–2014. Due to the high degree of missingness in measures of health system capacity, no imputations were conducted.

Data on the number of individuals living in urban areas were available by province for all years after 1994 from the General Statistics Office of Vietnam and are available online at the GSO website [37]. These data were used to calculate the proportion of the population in each province living in urban areas. Total population data was also available for all years after 1993 from the General Statistics Office of Vietnam. Missing population data were imputed linearly.

### Statistical analysis

Poisson-regression models were fit to provincial-level malaria case data from 1991 to 2014, using three different model outcomes: (1) clinically-diagnosed malaria cases (‘suspected’ cases), (2) blood-smear confirmed cases (‘confirmed’ cases), and (3) cases estimated by minimizing the year-to-year variation in the positive predictive value of clinical diagnosis, as described above (‘estimated’ cases). All case measures include cases of falciparum and vivax malaria. Models included a province-specific fixed effect and the log of the population as an offset term. Lagged variables were not used as the time stratification in the data is too coarse (see Peak et al. [24]). Models were fit using generalized estimating equations (GEE), appropriate for correlated time-series data, with a log-link function, an independent correlation structure, and a robust covariance matrix estimator [38]. Spearman’s rank correlation tests were conducted to investigate temporal trends in the data. Models were fit to data from all 51 provinces in Vietnam for the 24 years from 1991 to 2014. Additionally, provincial-level data were stratified into northern, central, and southern regions and models were separately fit in order to explore regional trends. Due to missingness in the health system capacity variables, a set of models was fit to data from 1997 to 2014 using all five covariates as predictors (i.e., ‘five-covariate models’) and another set of models was fit to data from 1991 to 2014 using only three covariates (i.e., ‘three-covariate models’), which excluded the two measures of health system capacity—discretionary budget and staff trainings. All analysis was conducted in R, using the *geepack* package for fitting generalized estimating equations [39].

## Results

### Changes in malaria transmission

**Fig. 1:**
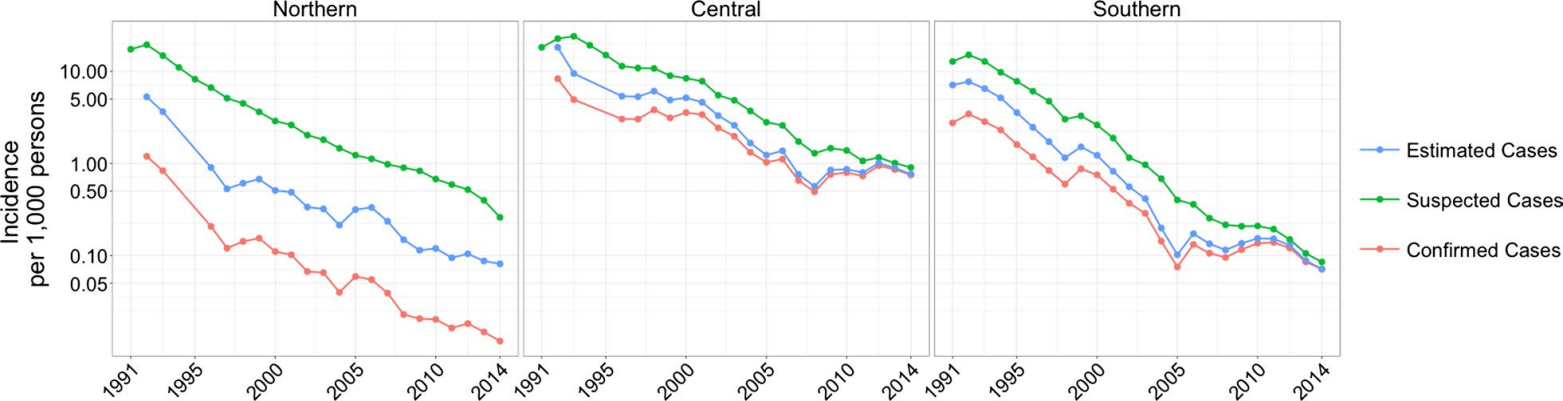
Incidence of suspected, confirmed, and estimated cases of malaria per 1000 person-years by region from 1991 to 2014 on a log-transformed scale where labels correspond to raw (unlogged) values. In 2014, there were 473 confirmed cases in the northern region, 12,006 confirmed cases in the central region, and 2,462 cases in the southern region

**Fig. 2:**
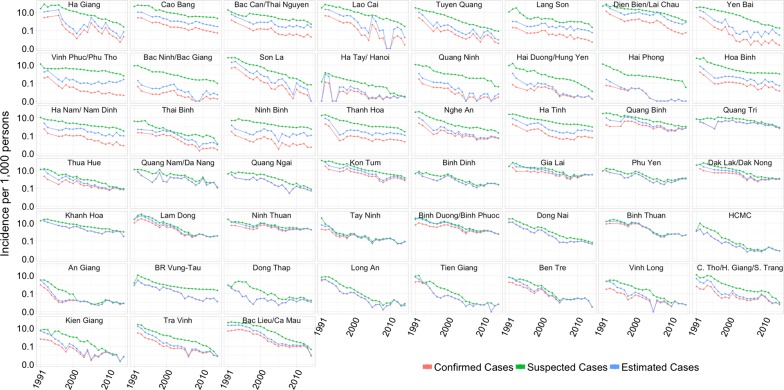
Incidence of suspected, confirmed, and estimated cases of malaria, by province (1992–2014). Provinces are arranged approximately by decreasing latitude (north to south) from top to bottom, and left to right. The *y*-axis is log-transformed, but the labels correspond to raw (unlogged) values and the “0.0” label on the *y*-axis corresponds to true zero

All measures of malaria incidence in Vietnam declined significantly between 1991 and 2014, with the majority of the decline occurring in the 1990s (Fig. 1). Suspected malaria cases in Vietnam declined from 1,290,250 cases in 1992 to 27,868 cases in 2014, corresponding to a 98.3% reduction in incidence. The confirmed case incidence decreased by 94.9% over this time period, from 224,923 cases in 1992 to 14,941 cases in 2014. Severe malaria cases declined from 24,022 cases in 1992 to 65 cases in 2014 and malaria fatalities declined from 2,702 deaths in 1992 to nine deaths in 2014. Similarly, estimated malaria cases declined 586,172 to 17,939 over this time. The declining trend in incidence between 1991 and 2014 was consistent across all provinces in Vietnam (Fig. 2).

### Malaria burden in Vietnam, 2014

**Fig. 3:**
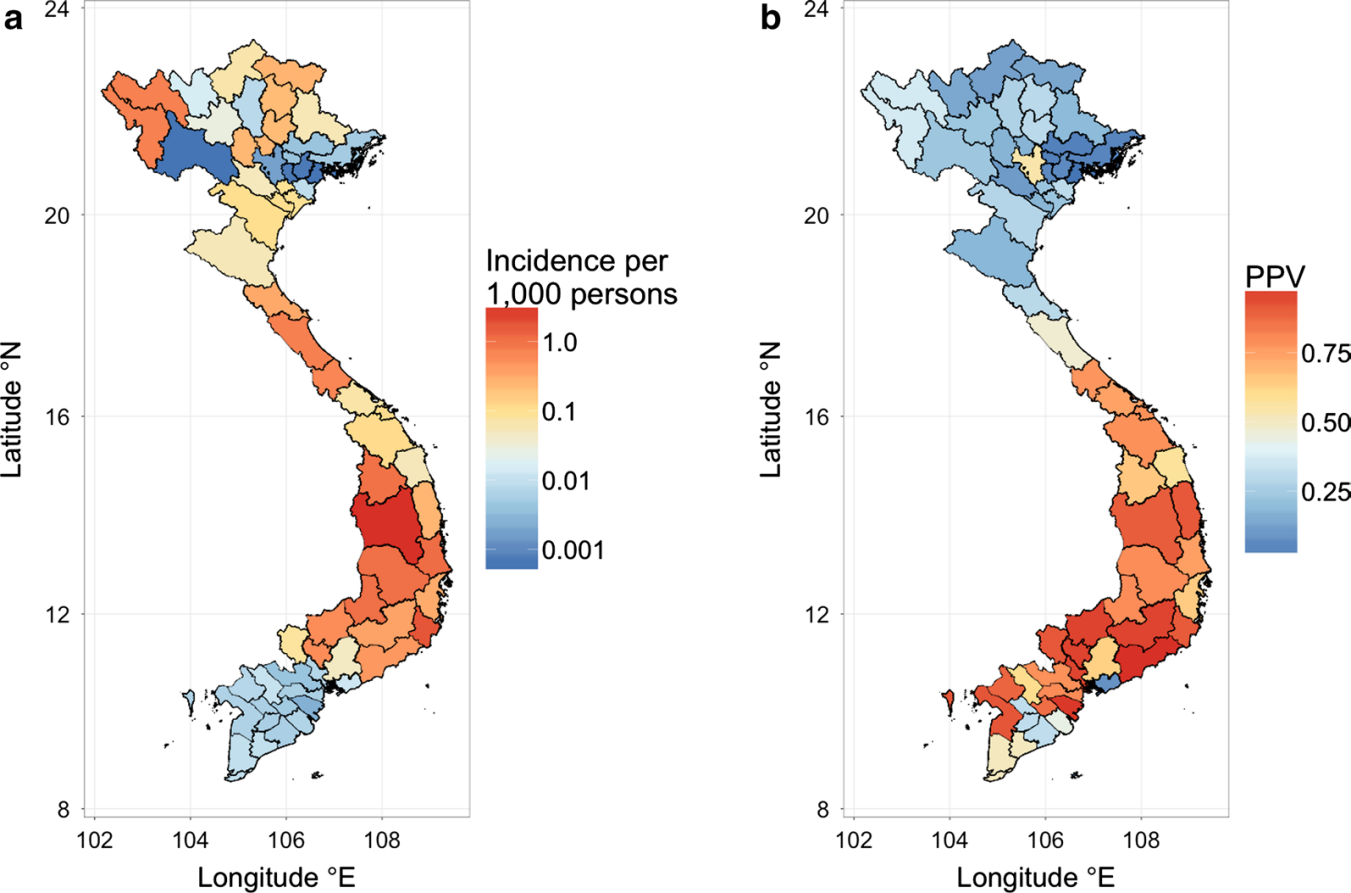
**a** Incidence of malaria in Vietnam, 2014. Incidence is calculated per 1000 person-years using the estimated number of cases. **b** Average positive predictive value, *q*, by province for the years 2010–2014

In 2014, malaria transmission was highest in Gia Lai province located in central Vietnam where an estimated 4,386 cases occurred. Only 22 provinces, most in central Vietnam, reported more than 50 confirmed malaria cases in 2014 (Table 1). Incidence varied greatly between northern provinces and was generally low in the southern provinces (Fig. 3a). Mapping the positive predictive value of clinical malaria diagnosis (*q*) in 2014 revealed that *q* tended to be much greater in the south than in the north (Fig. 3b), attributable to northern provinces having high numbers of suspected cases while reporting very few slide-confirmed cases. From 2010 to 2014, the average positive predictive values ranged from 0.10% in Hai Phong located in the northern region to 99.2% in Binh Thuan located in the southern region. Excluding the northern provinces and an outlier in Ba Ria-Vung Tau, the average positive predictive value ranged from 31.4% in Hau Giang/Can Tho/Soc Trang to 99.2% in Binh Thuan province; this is the expected range for the positive predictive value of malaria clinical diagnosis according to senior IMPE/NIMPE staff.

**Table 1:** Malaria cases in 2014, by province

Province name (latitude °N)	Estimated cases	Suspected cases	Confirmed cases	Incidence of estimated cases	Incidence of suspected cases	Incidence of confirmed cases
Dien Bien/Lai Chau (21.8°/22.4°)	700	1022	51	0.734	1.072	0.053
Thanh Hoa (20.1°)	374	957	69	0.107	0.274	0.020
Nghe An (19.2°)	163	604	144	0.054	0.199	0.047
Ha Tinh (18.3°)	408	1146	74	0.325	0.913	0.059
Quang Binh (17.5°)	647	930	596	0.745	1.071	0.686
Quang Tri (16.8°)	413	502	413	0.670	0.814	0.670
Thua Hue (16.5°)	80	103	78	0.071	0.091	0.069
Quang Nam/Da Nang (15.5°/16.0°)	283	331	283	0.114	0.133	0.114
Quang Ngai (15.1°)	64	99	64	0.052	0.080	0.052
Kon Tum (14.7°)	485	716	350	1.002	1.479	0.723
Binh Dinh (13.8°)	376	395	376	0.248	0.261	0.248
Gia Lai (13.8°)	4386	4424	4367	3.183	3.211	3.170
Phu Yen (13.1°)	983	1199	983	1.108	1.351	1.108
Dak Lak/Dak Nong (12.7°/12.3°)	2528	2921	2528	1.051	1.215	1.051
Khanh Hoa (12.3°)	376	1214	376	0.314	1.014	0.314
Lam Dong (11.9°)	465	479	465	0.369	0.38	0.369
Ninh Thuan (11.7°)	1033	1079	1033	1.750	1.828	1.750
Binh Duong/Binh Phuoc (11.3°/11.8°)	1600	1669	1600	0.570	0.595	0.570
Tay Ninh (11.4°)	90	98	90	0.082	0.089	0.082
Dong Nai (11.1°)	127	188	127	0.045	0.066	0.045
Binh Thuan (11.0°)	559	559	559	0.463	0.463	0.463
Ho Chi Minh City (10.8°)	57	57	57	0.007	0.007	0.007

Incidence measured per 1000 persons. Provinces ordered by decreasing latitude (°N) from north to south. Only the 22 provinces with > 50 suspected cases in 2014 are shown

### Malaria control measures between 1991 and 2014

**Fig. 4:**
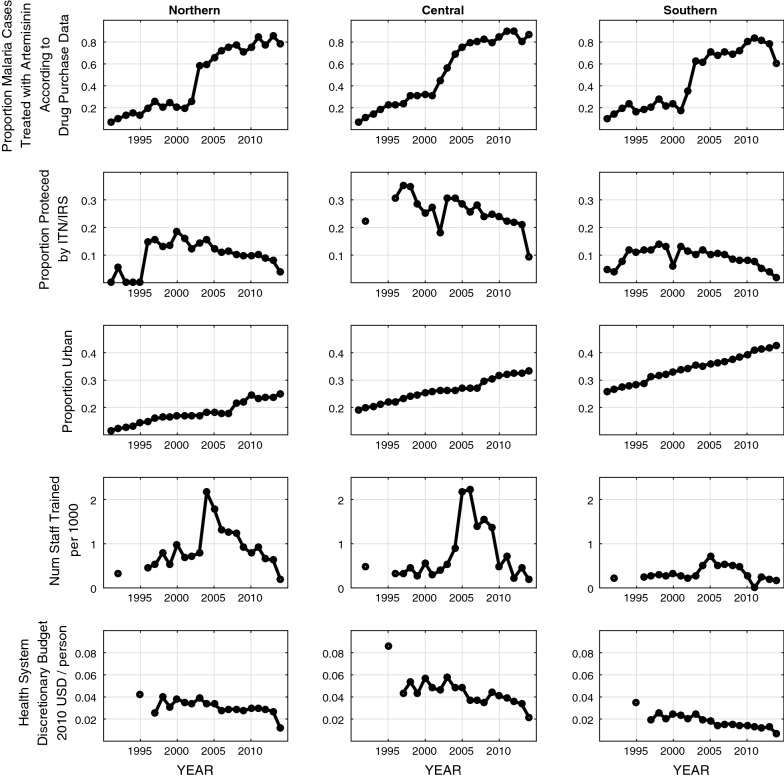
Changes in covariates between 1991 and 2014 for northern, central, and southern provinces

The percentage of treatment courses ordered for *P. falciparum* malaria that contained artemisinin increased significantly (Spearman $\rho$) from 12.1% in 1992 to 92.9% in 2014 across Vietnam and relatively uniformly for nearly all provinces (Fig. 4). The proportion of the population living in urban areas increased from 21.2% in 1995 to 33.1% in 2014. Again, this trend was similar across provinces and statistically significant almost everywhere. Unlike urbanization and the adoption of artemisinin, vector control measures did not show clear temporal trends when looking across provinces. Nationwide, the proportion of the population protected by vector control measures increased year-to-year from 1992 to 1997 (8.0% in 1992, 16.6% in 1996, 18.1% in 1997, 18.0% in 1998) and dropped to 9.7% in 2012 and 4.1% in 2014. Most provinces showed no temporal trend for vector control patterns.

### Effects of covariates on malaria incidence

**Fig. 5:**
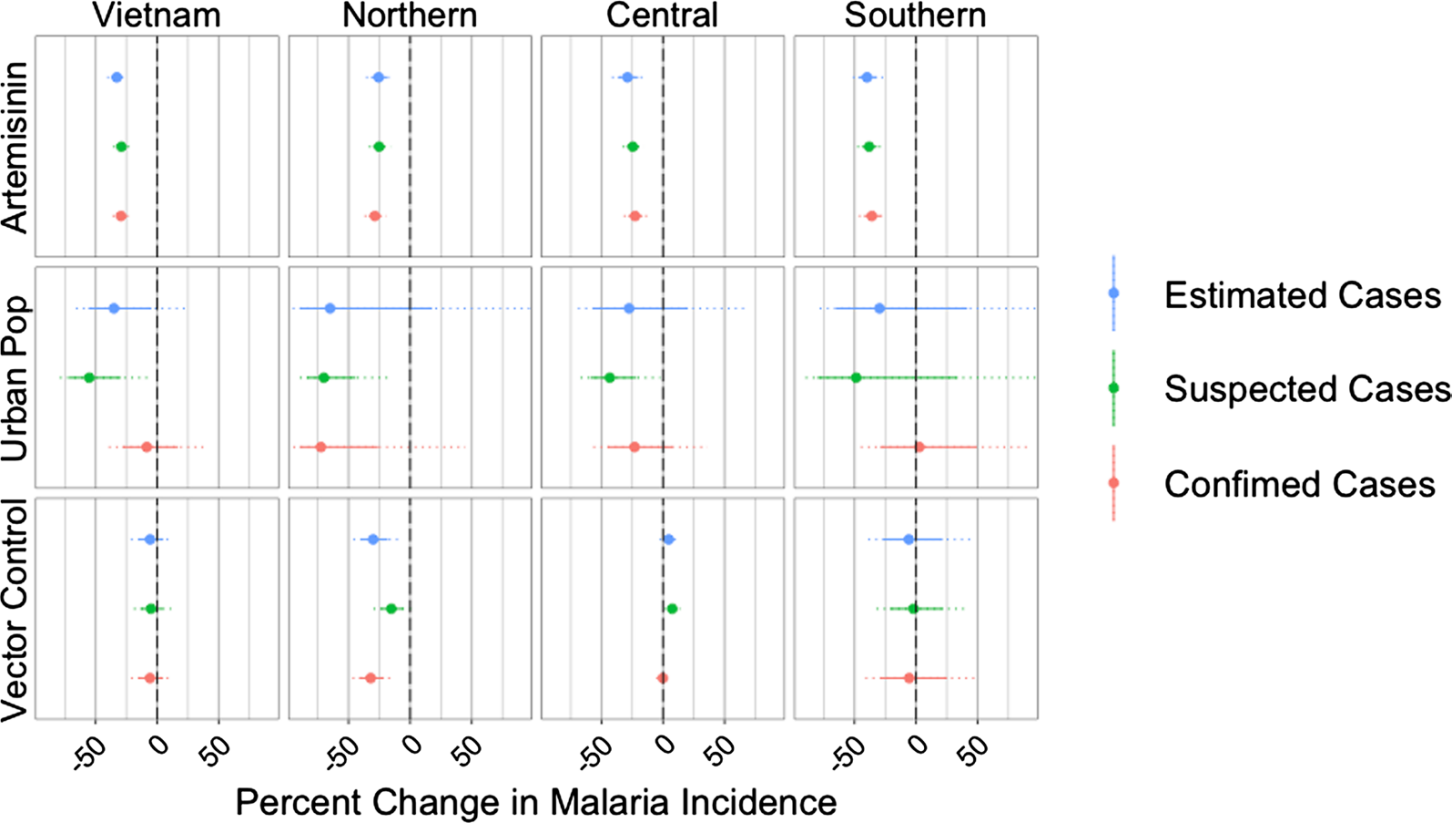
Percent change in malaria incidence associated with a 10% increase in the proportion of treatments for *P. falciparum* containing artemisinin (top row), proportion of the population living in urban areas (middle row), and proportion of the population protected by vector control measures (bottom row), by region and nationwide, as predicted by models using these three covariates only. The circle shows the mean effect size, the solid line shows the 95% confidence interval, and the dotted lines shows the 99.9% confidence interval. Outcome variable used in model is indicated by color. For clarity, the *x*-axis has been limited to range from – 90 to 90%

In the analysis using all 51 provinces, after controlling for the proportion of the population living in urban areas and the proportion of the population protected by vector control, the proportion of treatments for *P. falciparum* that contained artemisinin was significantly (*p* < 0.001) and inversely associated with all three measures of malaria incidence (Fig. 5). A 10% increase in the proportion of treatments containing artemisinin was associated with a 32.8% (95% CI 27.7–37.5%) reduction in the incidence of estimated cases, a 29.4% (95% CI 24.9–33.5%) reduction in the incidence of confirmed cases, and a 29.0% (95% CI 24.7–33.1%) reduction in the incidence of suspected cases. The proportion of the population living in urban areas was significantly and inversely associated with suspected (*p* < 0.001) and estimated (*p* < 0.05) cases, but not with confirmed cases. The proportion of the population protected by vector control measures was not significantly associated with any of the three measures of malaria incidence in the nationwide analysis.

**Fig. 6:**
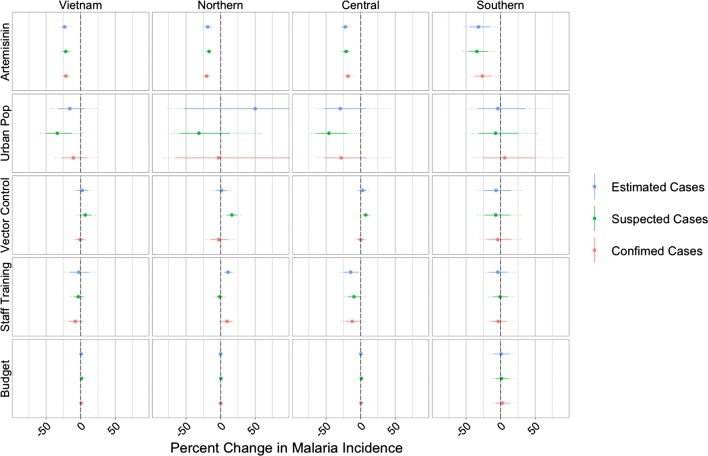
As in Fig. 5, with the addition of covariates measuring health system capacity

Additionally controlling for changes in health system capacity using staff trainings and the discretionary budget similarly revealed that the proportion of treatments for *P. falciparum* containing artemisinin was significantly (*p* < 0.001) inversely associated with incidence, as measured by suspected, confirmed, and estimated cases (Fig. 6). Again, no significant associations were found between the proportion of the population protected by vector control and any of the three measures of malaria incidence. The proportion of the population living in urban areas was significantly inversely associated with suspected cases (*p* = 0.004), but was not significantly associated with estimated or confirmed cases. The discretionary budget was found to be positively but weakly associated with the number of suspected cases, with a 10% budget increase corresponding to a 1.35% (95% CI 0.09–2.6%) increase in the number of suspected cases (*p* = 0.035). Staff trainings per 100-persons was not found to be significantly associated with any incidence measure.

### Regional variation

The proportion of purchased *P. falciparum* treatments that contained artemisinin was found to be significantly (*p* < 0.001) inversely associated with all three measures of malaria incidence in all three regions (northern, central, southern) of Vietnam, both with (Fig. 5) and without (Fig. 6) the inclusion of the health system capacity variables. A possible inverse association between the proportion of the population living in urban areas and malaria incidence can be seen for the central provinces, although it is only significant when looking at suspected cases. The relationship between urbanization and malaria in the northern provinces is difficult to assess as the inferred associations were not robust across models and covariates. There was no evidence of a relationship between urbanization and malaria in the southern provinces.

Although the proportion of the population protected by vector control measures was not found to be significantly associated with malaria incidence when all provinces where analysed together, a few significant associations were identified when the analysis was stratified by region. In the three-covariate models, the proportion protected by vector control measures was significantly and inversely associated with all three measures of malaria incidence in the northern provinces (*p* < 0.01). However, in the five-covariate models, this association only remained significant for incidence measured by suspected cases and the directionality of the effect was reversed. For the southern and central provinces, no statistical evidence to support an association between vector control and malaria incidence was found. The lack of robustness in these results and the presence of positive associations between vector control and malaria suggested that the NIMPE/IMPE data did not contain any evidence for an effect of vector control on malaria incidence in Vietnam.

The discretionary budget was not significantly associated with any of the three measures of incidence in any of the three regions. Staff trainings were significantly and inversely associated with confirmed and estimated (*p* < 0.05) cases in the central region; however, in the northern region, staff trainings were positively associated with confirmed (*p* < 0.01) and estimated ( *p*< 0.001) cases. Overall, the health capacity variables do not have robust associations with malaria incidence; it is important to remember that as malaria incidence drops, budgets and staff numbers for malaria control programmes will also be reduced.

The results of the entire analysis were robust to the method of calculation for the proportion of treatments for *P. falciparum* containing artemisinin (see Additional file 3).

## Discussion

The most robust statistical association in the data reported by Vietnam’s National Institutes for Malariology, Parasitology, and Entomology from 1991 to 2014 is the negative association between malaria incidence—measured in three different ways—and the purchases of artemisinin-based drugs (as a fraction of total drug purchases) by provincial malaria control programmes. The significant negative association between the proportion of treatments for *P. falciparum* containing artemisinin and malaria incidence persists whether or not health system variables are included in the analysis and whether the data are analysed regionally or nationally. These findings are consistent with those of Peak et al. [24] in their analysis of southern Vietnam from 1991 to 2010.

### Data limitations

Although the predictor data assembled for this study only show that artemisinin-derivatives were purchased (but not used or prescribed), other descriptions of the health system and anti-malarial usage in Vietnam in the 1990s indicate that artemisinin drugs were in fact used and probably favoured, when compared with other drugs, in the treatment of *P. falciparum* malaria [33, 34]. In 2003, anti-malarial treatment guidelines were updated in Vietnam to reflect the fact that artemisinin therapies had been accepted worldwide as the most effective treatment for uncomplicated falciparum malaria; indeed, it was the trials conducted in southern Vietnam in the 1990s [4, 5] that initiated these discussions. Non-artemisinin therapies were no longer recommended after 2003 in Vietnam, and the average clinician’s familiarity and experience with artemisinin-based therapies would have meant these drugs were favoured as treatment for malaria. Malaria case numbers were already quite low in 2003 (61,204 estimated cases) and the NIMPE data indicate that sufficient numbers of courses were available for all provinces. Clinicians working in southern Vietnam at the time would estimate that the vast majority of falciparum malaria cases would have received an artemisinin-containing therapy as first-line treatment. Nevertheless, as systematic data on usage or prescription are not available in the NIMPE reports, this is a limitation in the present analysis and any analysis linking drug/treatment purchase data to incidence.

A second limitation of the data is the lack of coverage information. As anti-malarial drugs in Vietnam have been free at least since the 1990s, the question of coverage reduces to a question of access and education. Publicly available demographic health surveys (1997, 2002, 2005) do not contain information on the percentage of children or adults who took an anti-malarial for a febrile episode suspected of being malaria. As with the question on drug purchases and drug use, with no direct coverage data available, the best source of information comes from clinicians with local knowledge of access, treatment-seeking habits, and the current malaria burden. In 2014, it is very likely that treatment coverage for a febrile malaria episode was 100%. It is also likely that, since the turn of century, treatment coverage was very high or near 100%. During the 1990s, it is not possible to make an educated guess on how high treatment coverage levels were. Systematically collected data on coverage do not exist, and this analysis does not aim to test whether treatment coverage was an influential covariate in reducing malaria case counts in Vietnam from 1991 to 2014.

Additional general limitations of this analysis include the reporting system itself as it does not include true positives that did not report to the health system. Individuals with mild symptoms are less likely to seek care and, when they do, are more likely to receive a false negative clinical diagnosis. These cases are known to occur in Vietnam [40], but it is unlikely that they represent a major proportion of the population. Second, the coarse nature of the data (annual aggregation) reduces certainty in associations and prevents observation of short-term effects. A cohort with active surveillance and knowledge on anti-malarial drug use and other interventions would be the ideal data set for inferring these associations. These study designs are common in Africa where transmission levels are still high, but the low number of malaria cases in Vietnam makes such studies impractical.

### Comparisons across regions

The results of this study differ substantially from a recent continent-wide analysis in Africa showing that approximately 68% of the decline in malaria from 2000 to 2015 can be attributed to the use of insecticide-treated nets (ITNs) [1]. One reason may be the differential effort in Vietnam placed on ensuring access to ACT versus distributing ITNs. ACT medicines in Vietnam are free in the public sector and there are virtually no private sector sales. With low annual case numbers and a concentration of cases in a few provinces, Vietnam has achieved nearly full ACT coverage for malaria, while implementation of vector control is irregular and generally reaches < 30% of the population in endemic provinces. This contrasts with the access and treatment scenario in Africa where it was recently reported that only 20% of children under the age of five received an ACT for a confirmed case of *P. falciparum* malaria [41]. As ACT scale-up from 2000 to 2015 reached only 20% coverage in Africa, while ITN scale-up reached coverages between 40 and 70% [42], perhaps it is not surprising that the estimated effect size of ACT on malaria incidence in Africa is low. In principle, a statistical effect should be detectable for small increases of ACT coverage, but in practice these effects tend to be non-linear and an increase from 0 to 20% may not have the same effect as an increase from 20 to 40%. The differing results between Vietnam and Africa are not necessarily contradictory. It may simply be the case that in Africa we have not yet had an opportunity to fully measure the extent to which ACT could reduce malaria incidence under a scenario of widespread access to ACT.

A second potential explanation for differences in ITN/IRS efficacy across continents may lie in the biting habits of the most common *Anopheles* species in each region. Three of the most common vector species in central Vietnam (where the majority of malaria transmission occurs today) are *Anopheles dirus*, *Anopheles maculatus*, and *Anopheles minimus*. While *An. maculatus* appears to bite outdoors and early in the evening (making ITN use less effective), biting behaviour varies substantially for *An. dirus* and *An. minimus* [43]. *Anopheles gambiae* and *Anopheles funestus* are the most common species in west and central Africa and their feeding and resting habits inside and outside households have been described in numerous studies over the years. Nevertheless, changes through time in anopheline species distributions and feeding habits (due to ITN use or IRS) make it impossible to specify whether vector control measures should be effective based on species distribution alone. Exact feeding habits for each species differ from region to region and frequently depend on past insecticide and bed net use [44], making it difficult to compare large geographic areas on their potential for successful vector-based intervention.

A third possible explanation for the differing conclusions reached using Vietnamese data and African data is that in low transmission regions, ACT case management is more effective than ITN use as a general malaria control policy, whereas the reverse may be true for high transmission regions. ACT case management is a control strategy that works by targeting symptomatic individuals, while vector control acts broadly to protect the entire at-risk population. As a result, ACT case management may be less effective at reducing transmission in highly endemic areas due to the presence of asymptomatic cases that may never be diagnosed and treated [30, 45]. In Vietnam, transmission intensity and population immunity are low, and individuals with malaria are more likely to experience symptoms, seek treatment, and receive an ACT [46, 47]. The population-level effects of ACT case management in areas of low transmission suggest that rapid ACT scale-up could be an effective endgame strategy for regions close to achieving elimination [30]. Additionally, bed nets may have played a less important role in the decline of malaria in Vietnam between 1991 and 2014 due to the challenge of increasing bed net utilization in specific high-risk groups such as forest workers who stay overnight in areas where transmission intensity is the greatest [48].

### Positive predictive value

The positive predictive value (PPV) of malaria clinical diagnosis revealed that the average PPV for the years 2010–2014 in the northern part of the country ranged from 0.10 to 54.44%, whereas in the central and southern parts of the country, PPV ranged from 31.4 to 99.2%. In the northern provinces, suspected case counts are high, but confirmed case counts are low. Based on Vietnam’s substantial experience with malaria microscopy and clinical malaria diagnosis, and the existence of a centralized malaria health system, it is likely that over-reporting of suspected malaria cases is occurring in the northern provinces. It is much less likely that a lack of microscopes, microscopists, or a truly low PPV of clinical diagnosis is the cause of the large number of suspected cases reported from the north.

### Outlook

A key evaluation that will need to be made in the coming years is whether the use of ACT in Vietnam continues to be associated with declining malaria in the presence of drug resistance. Mutations in the kelch protein of *P. falciparum* have been shown to be associated with slower clearance of parasites by the artemisinin derivatives [49]. These mutations were first seen at appreciable frequency in Binh Phuoc province in Vietnam during the last 4 months of 2014 [50], and they should be monitored in conjunction with absolute case counts to determine if additional control efforts are needed due to failed treatments and sustained incidence.

With communicable diseases still playing a large role in the World Health Organization’s health-related Sustainable Development Goals for 2030, malaria elimination will stay on the agenda as an important public health priority in countries that are in or approaching near-elimination phase. Vietnam reported fewer than 10,000 confirmed malaria cases both in 2015 and 2016, placing it in a small group of 30–35 countries that could realistically eliminate malaria by 2030 [51]. As monitoring and active surveillance scale up during this phase, it is critical to understand the local causes of malaria decline over the past ten or more years that have enabled each country to reach near-elimination phase. In Vietnam, the path to low malaria incidence has clearly been led by high levels of artemisinin and ACT use in the public sector at coverage rates that can realistically be considered as having a noticeable impact on malaria elimination in some provinces. Active surveillance, reactive case detection, and following at-risk groups are the next key focal areas in Vietnam’s next phase of moving the majority of its provinces to zero malaria over the next decade. The major gloom on the horizon is the spread of artemisinin-resistant genotypes in Vietnam [50, 52, 53], as the arrival of drug-resistance can undermine elimination efforts [54, 55]. It is uncertain if a public health response in this context (e.g., lengthening ACT courses, follow-up with second-line drugs) will be sufficient to maintain the cure rates previously observed with ACT and keep Vietnam on the road to elimination. Public health agencies and researchers must work together during this time to share knowledge and data, remain open to quick changes in public health strategy, and squarely keep the focus on the public good of eliminating malaria.

## Additional files

**Additional file 1.** Regional groupings of provinces used in the analysis.

**Additional file 2.** Additional methods and tables.

**Additional file 3.** Regression results using the alternative calculation for the proportion of treatments containing artemisinin.
