# Supplementary material for: The decline of malaria in Vietnam, 1991–2014
Source: Malar J. 2018 Jun 7;17:226. doi: 10.1186/s12936-018-2372-8 (PMC5992833; doi:10.1186/s12936-018-2372-8)
Supplement: Supplementary file 1 — Additional file 1. Regional groupings of provinces used in the analysis. [file 12936_2018_2372_MOESM1_ESM.pdf]

Additional File 1

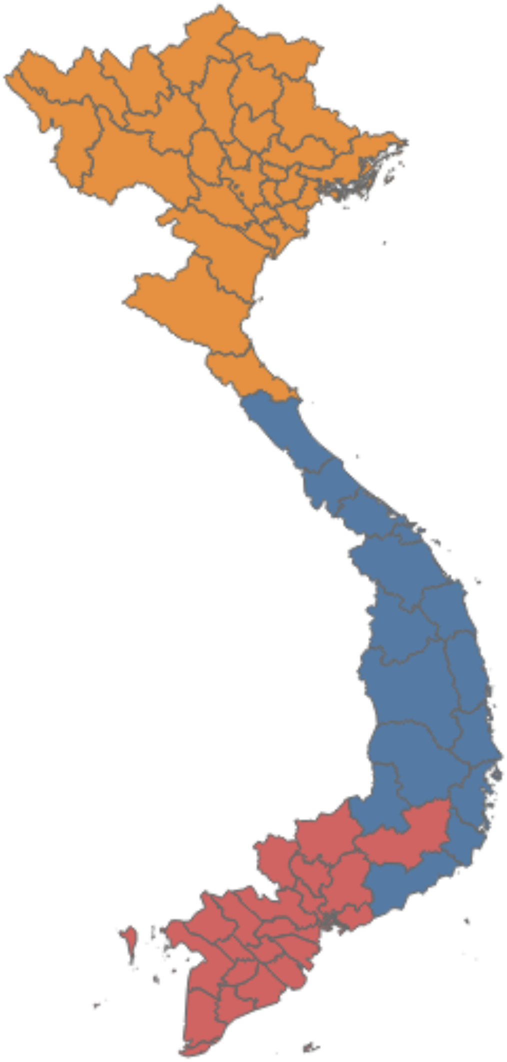

Note. Northern (orange), central (blue), and southern (red) regional grouping of provinces used in the analysis.
