# Supplementary material for: The decline of malaria in Vietnam, 1991–2014
Source: Malar J. 2018 Jun 7;17:226. doi: 10.1186/s12936-018-2372-8 (PMC5992833; doi:10.1186/s12936-018-2372-8)
Supplement: Supplementary file 2 — Additional file 2. Additional methods and tables. [file 12936_2018_2372_MOESM2_ESM.pdf]

Additional File 2

Data on the use of anti-malarial drugs were contained in these reports in the form of requests placed by provincial malaria control programmes to NIMPE for anti-malarial drugs based on projected need for the subsequent year (Table S1). Requests for anti-malarial drugs likely capture true anti-malarial drug usage in the country because all public hospitals and health centers in Vietnam provide anti-malarial drugs free of charge to individuals suspected of having malaria [24, 35]. This study looked at treatments for falciparum malaria only because the vast majority of suspected malaria cases were not confirmed by blood-smear for the first half of the time series, and unconfirmed cases were typically presumed to be *P. falciparum* and treated as such. In a setting where all suspected malaria is confirmed as *P. falciparum* or *P. vivax*, artemisinin would only act to reduce falciparum transmission; however, when the majority of cases are unconfirmed, the presumption of falciparum and treatment with artemisinins will act to reduce onward transmission of both falciparum and vivax. Treatment guidelines used to convert drug units ordered to drug courses ordered are shown in Table S2.

Table S1

Anti-malarial drugs available in Vietnam, 1991-2014

Drug	Unit Weight	Years Requested
DHA-PPQ	40 mg DHA/ 320 mg Piperaquine	2007-2014
CV8	32 mg DHA/ 320mg PPQ/ 90 mg trimethoprim/ 5 mg primaquine	2001-2006
Artemisinin	250 mg	1992-2003
Artesunate	50 mg	1992-2008
Artesunate Injection	60 mg	1994-2014
Quinine sulfate (oral use)	250 mg	1992-2001; 2004-2005; 2007-2008; 2011-2014
Quinine hydrochloride (IM injection)	500 mg	1992-2001; 2004-2005
Quinine dihydrochloride (IV injection)	500 mg	1992-1995
Primaquine	13.2 mg	1992-2014
Chloroquine	250 mg	1992-2014
Mefloquine	250 mg	1994-1997; 1999- 2002
SR2	500 mg Sulfadoxine/ 25 mg Pyrimethamine	1992-2003
SR3	combination therapy based on Sulfadoxine or Sulfadimethoxin (rarely ordered, and only in 1992)	1992
Fansidar Injection	400mg Sulfadoxine/ 20mg Pyrimethamine 2.0 mL	1995

Note. DHA = Dihydroartemisinin; IM = Intramuscular; IV = Intravenous

Additional File 2 (continued)

Table S2

Treatment Guidelines for Malaria in Adults

Guidelines prior to 2003	Drug (Avg. units per total treatment course)
Uncomplicated *P. falciparum*	Chloroquine (8 tablets) + Primaquine (4 tablets)
Uncomplicated *P. falciparum* (with chloroquine resistance)	Fansidar (3 tubes)
	OR Fansidar (3 tubes) + Quinine (42 tablets)
	OR Quinine (42 tablets) + Tetracycline (28 tablets)
	OR Mefloquine (3 tablets)
	OR Artemisinin (16 tablets, 7 days) + Primaquine (4 tablets)
Severe *P. falciparum*	Artesunate Injection (4-8 tubes)
	OR Quinine hydrochloride (IM route) (21 tubes)
P. vivax	Chloroquine (8 tablets) + Primaquine (20 tablets)
Guidelines issued June 2003 and January 2007	
Uncomplicated *P. falciparum*	Artesunate (16 tablets) + Primaquine (4 tablets)
	OR CV8 (8 tablets) OR DHA-PPQ (8 tablets)
Uncomplicated *P. falciparum* (in 1st trimester pregnancy)	Quinine (42 tablets)
Severe *P. falciparum*	Artesunate Injection (2-9 tubes with switch to oral)
P. vivax	Chloroquine (10 tablets) + Primaquine (20 tablets)
Guidelines issued November 2009	
Uncomplicated *P. falciparum*	DHA-Piperaquine (8 tablets) + Primaquine (4 tablets)
Uncomplicated *P. falciparum* (in 1st trimester pregnancy)	Quinine (42 tablets) + Clindamycin (14 tablets)
Severe *P. falciparum*	Artesunate Injection (4-10 tubes with switch to oral)
P. vivax	Chloroquine (10 tablets) + Primaquine (28 tablets)
