# Supplementary material for: The decline of malaria in Vietnam, 1991–2014
Source: Malar J. 2018 Jun 7;17:226. doi: 10.1186/s12936-018-2372-8 (PMC5992833; doi:10.1186/s12936-018-2372-8)
Supplement: Supplementary file 3 — Additional file 3. Regression results using the alternative calculation for the proportion of treatments containing artemisinin. [file 12936_2018_2372_MOESM3_ESM.pdf]

Additional File 3

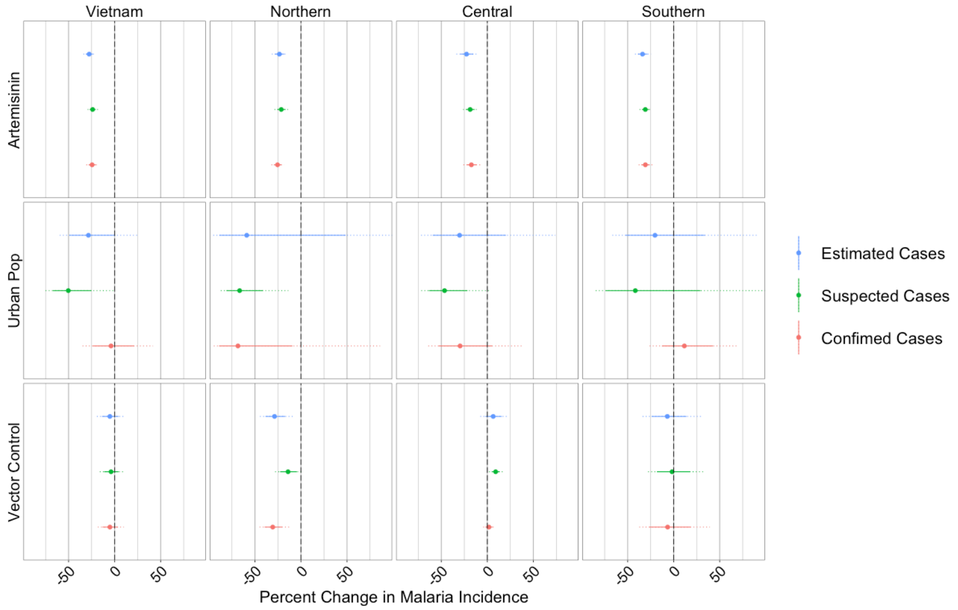

(a) Three-covariate models

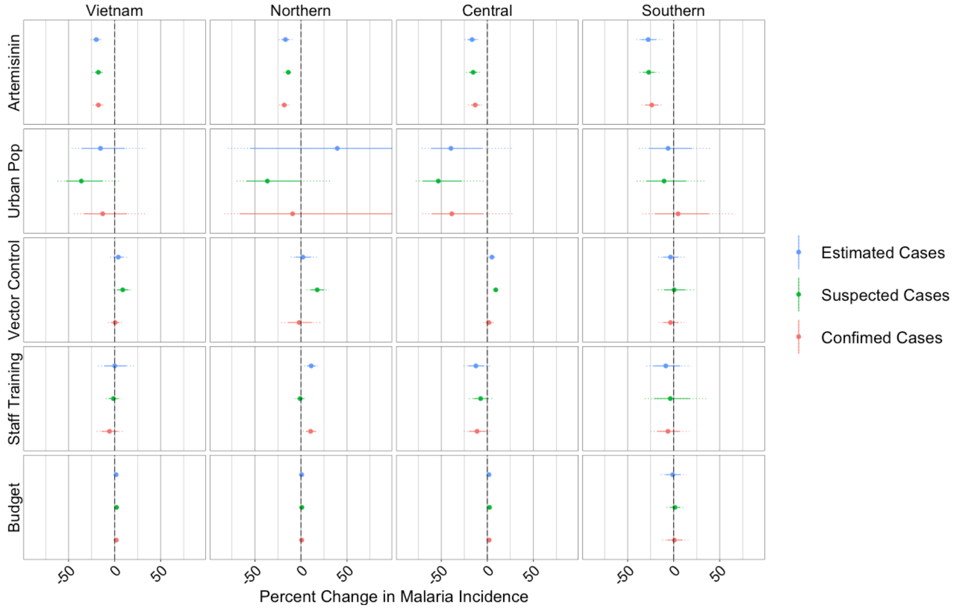

(b) Five-covariate models

Note. Regression results using the alternative calculation for the proportion of treatments containing artemisinin, shown as the percent change in incidence associated with a 10% increase in the listed covariate, by region and for all provinces in Vietnam for **a)** the models including three covariates only, and for **b)** the models that included two additional covariates as measures of health system capacity. The circles show the mean effect sizes, the solid lines show the 95% confidence intervals, and the dotted lines show the 99.9% confidence intervals. Model outcome (estimated, suspected, or confirmed cases) is indicated by effect size color. For clarity, the x-axis has been limited to range from -90 to 90.
